# Supplementary material for: Innervation density governs crosstalk of GPCR-based norepinephrine and dopamine sensors
Source: bioRxiv. 2024 Nov 23:2024.11.23.624963. Preprint. [Version 1] doi: 10.1101/2024.11.23.624963 (PMC11601633; doi:10.1101/2024.11.23.624963)
Supplement: 1 [file NIHPP2024.11.23.624963V1-supplement-1.pdf]

bioRxiv preprint doi: https://doi.org/10.1101/2024.11.23.624963; this version posted November 23, 2024. The copyright holder for this preprint (which was not certified by peer review) is the author/funder, who has granted bioRxiv a license to display the preprint in perpetuity. It is made available under aCC-BY 4.0 International license.

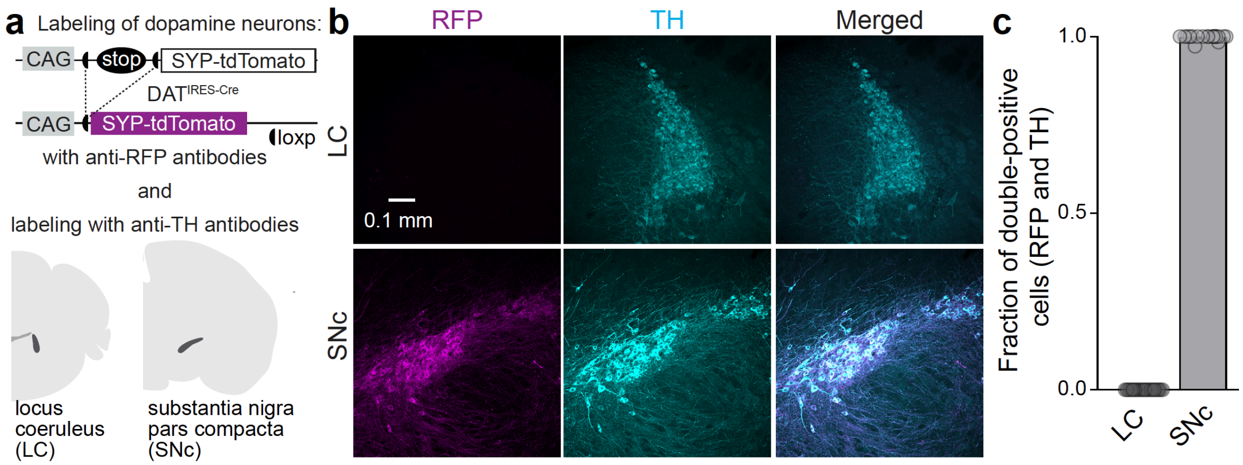

Supplemental figure 1. Assessment of Cre-dependent expression of synaptophysin-tdTomato in DAT$^{IRES\text{-}Cre}$ mice

a. Strategy for labeling dopamine and norepinephrine neurons similar to Fig. 1a-c but with staining for tyrosine hydroxylase (TH) followed by analyses of immunofluorescence in locus coeruleus (LC) and substantia nigra pars compacta (SNc).

b, c. Representative confocal images of LC and SNc (b) and quantification of the fraction of cells double positive for RFP and TH (c); LC 20 slices from 4 mice, SNc 20/4.

Data are mean ± SEM. On average, 96 ± 6 cells in LC and 88 ± 6 cells in SNc were positive for TH per image. The observation that there are no double-positive cells in LC indicates that the DAT$^{IRES\text{-}Cre}$ line does not express Cre in LC neurons. Hence, the strategy of NET labeling combined with RFP-labeling of SYP-tdTomato in DAT$^{IRES\text{-}Cre}$ mice can be used to distinguish norepinephrine from dopamine axons. Both norepinephrine and dopamine neurons express TH.

bioRxiv preprint doi: https://doi.org/10.1101/2024.11.23.624963; this version posted November 23, 2024. The copyright holder for this preprint (which was not certified by peer review) is the author/funder, who has granted bioRxiv a license to display the preprint in perpetuity. It is made available under aCC-BY 4.0 International license.

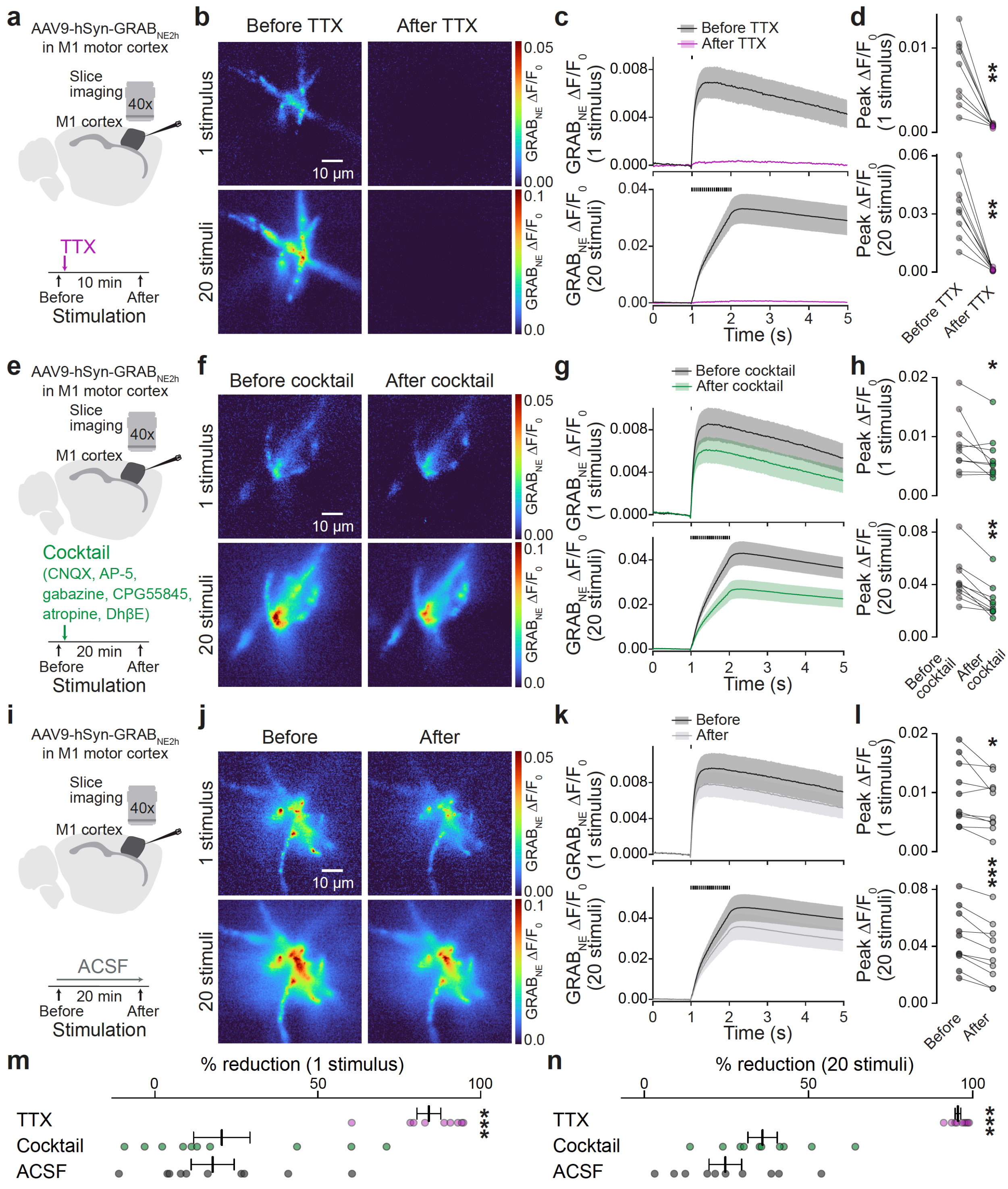

Supplemental figure 2. Pharmacological inhibition of $GRAB_{NE}$ transients in brain slices

a. Schematic of imaging in cortex before and after 10-minute bath application of the sodium channel blocker tetrodotoxin (TTX) to inhibit action potential firing.

bioRxiv preprint doi: https://doi.org/10.1101/2024.11.23.624963; this version posted November 23, 2024. The copyright holder for this preprint (which was not certified by peer review) is the author/funder, who has granted bioRxiv a license to display the preprint in perpetuity. It is made available under aCC-BY 4.0 International license.

b-d. Representative images of peak $GRAB_{NE}$ fluorescence in response to 1 stimulus or 20 stimuli at 20 Hz before and after TTX wash-on (b) and quantification of $GRAB_{NE}$ $\Delta F/F_0$ time series (c) and peak $\Delta F/F_0$ (d); 9 slices from 3 mice.

e-h. Same as in a-d, but with bath application of a drug cocktail containing CNQX, AP-5, gabazine, CGP-55845, atropine, and DHβE for 20 min; 10/3.

i-l. Same as in a-d, but with incubation in ACSF for 20 min; 10/3.

m, n. Post-hoc assessment of percent reduction in response to 1 stimulus (m) or 20 stimuli (n) comparing incubation in TTX (a-d, 10 min), the drug cocktail (e-h, 20 min), or ACSF (i-l, 20 min); TTX 9/3, cocktail 10/3, and ACSF 10/3. Overall, rundown during drug cocktail application and incubation in ACSF was similar while TTX strongly inhibited the fluorescence changes.

Data are mean ± SEM; *** $p < 0.001$, ** $p < 0.01$, * $p < 0.05$, as assessed by: two-tailed Wilcoxon signed-rank tests in d and h; two-tailed paired t-tests in l; one-way ANOVA and Dunnet's multiple comparisons post-hoc tests in m (compared to ACSF); Kruskal-Wallis and Dunn's multiple comparisons tests in n (compared to ACSF).

bioRxiv preprint doi: https://doi.org/10.1101/2024.11.23.624963; this version posted November 23, 2024. The copyright holder for this preprint (which was not certified by peer review) is the author/funder, who has granted bioRxiv a license to display the preprint in perpetuity. It is made available under aCC-BY 4.0 International license.

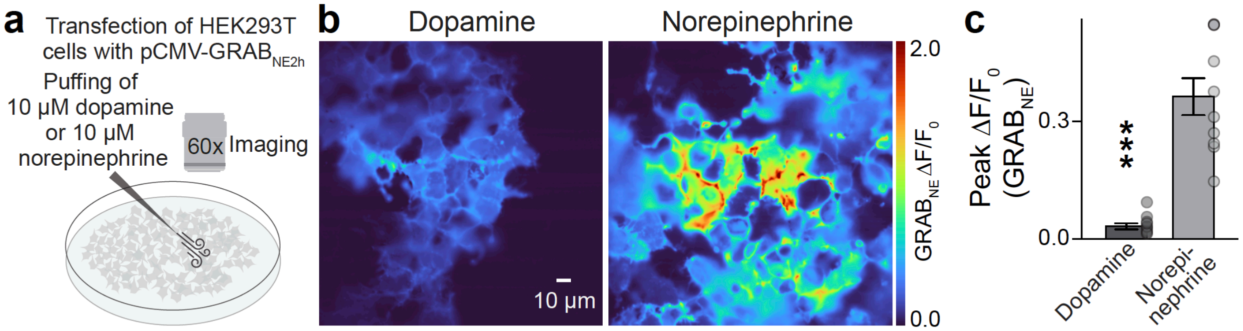

Supplemental figure 3. Assessment of GRAB$_{NE}$ fluorescence in HEK293T cells

a. Schematic of the experiment in which 10 μM of dopamine or norepinephrine were puffed onto HEK293T cells transfected with pCMV-GRAB$_{NE2h}$.

b, c. Representative peak GRAB$_{NE}$ fluorescence (b) and peak $\Delta F/F_0$ (c); 10 coverslips from 3 transfections each.

Data are mean ± SEM; *** $p < 0.001$, as assessed by two-tailed Mann-Whitney rank-sum test in c.

bioRxiv preprint doi: https://doi.org/10.1101/2024.11.23.624963; this version posted November 23, 2024. The copyright holder for this preprint (which was not certified by peer review) is the author/funder, who has granted bioRxiv a license to display the preprint in perpetuity. It is made available under aCC-BY 4.0 International license.

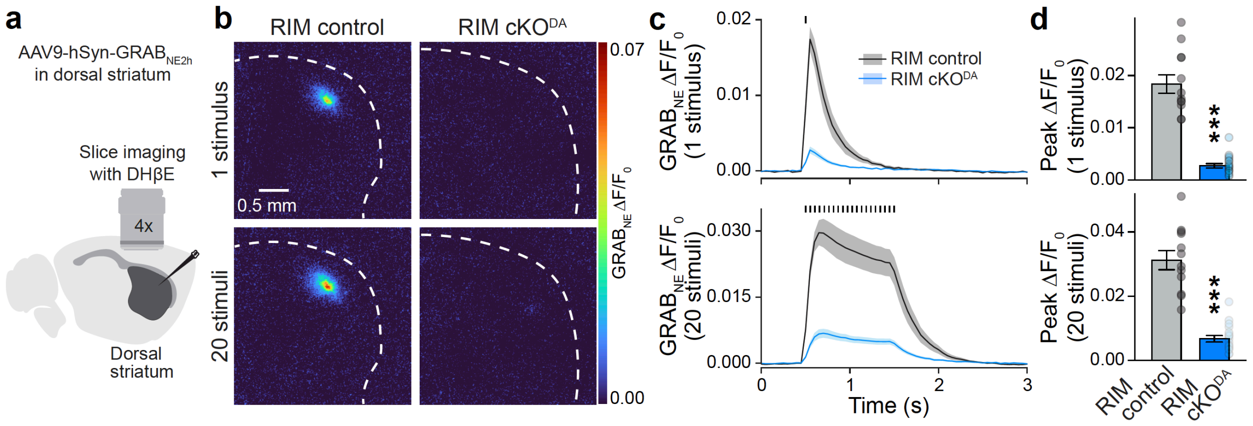

Supplemental figure 4. GRAB$_{NE}$ recordings in striatum in the presence of DHβE

a. Schematic of imaging experiments as in Fig. 2c-e, but in the presence of dihydro-β-erythroidine hydrobromide (DHβE, 1 μM) to block β2-containing nicotinic acetylcholine receptors.

b-d. Representative images of peak GRAB$_{NE}$ fluorescence in response to 1 stimulus or 20 stimuli at 20 Hz (b) and quantification of GRAB$_{NE}$ $\Delta F/F_0$ time series (c) and peak $\Delta F/F_0$ (d); RIM control 12 slices from 3 mice, RIM cKO^{DA} 17/4.

Data are mean ± SEM; *** $p < 0.001$, as assessed by two-tailed Mann-Whitney rank-sum tests in d.

bioRxiv preprint doi: https://doi.org/10.1101/2024.11.23.624963; this version posted November 23, 2024. The copyright holder for this preprint (which was not certified by peer review) is the author/funder, who has granted bioRxiv a license to display the preprint in perpetuity. It is made available under aCC-BY 4.0 International license.

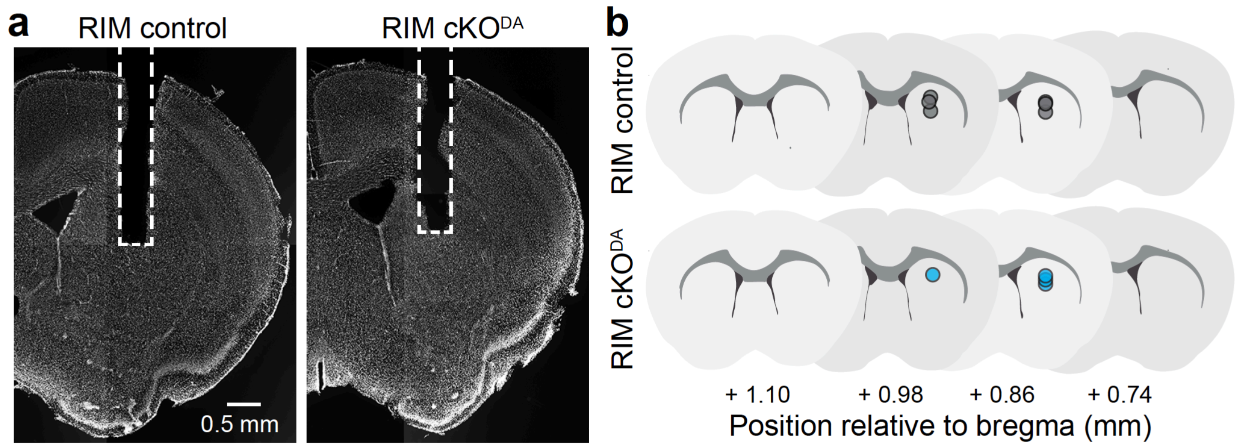

Supplemental figure 5. Fiberoptic cannula positions

a. Representative images of coronal sections of mice with fiberoptic cannulas. Coronal sections were imaged with a slide scanner in DAPI-containing mounting medium.

b. Illustration of fiberoptic cannula tip positions of the mice analyzed in Fig. 2k determined relative to a mouse brain atlas[38] and mapped onto schematics drawn from brain sections.

bioRxiv preprint doi: https://doi.org/10.1101/2024.11.23.624963; this version posted November 23, 2024. The copyright holder for this preprint (which was not certified by peer review) is the author/funder, who has granted bioRxiv a license to display the preprint in perpetuity. It is made available under aCC-BY 4.0 International license.

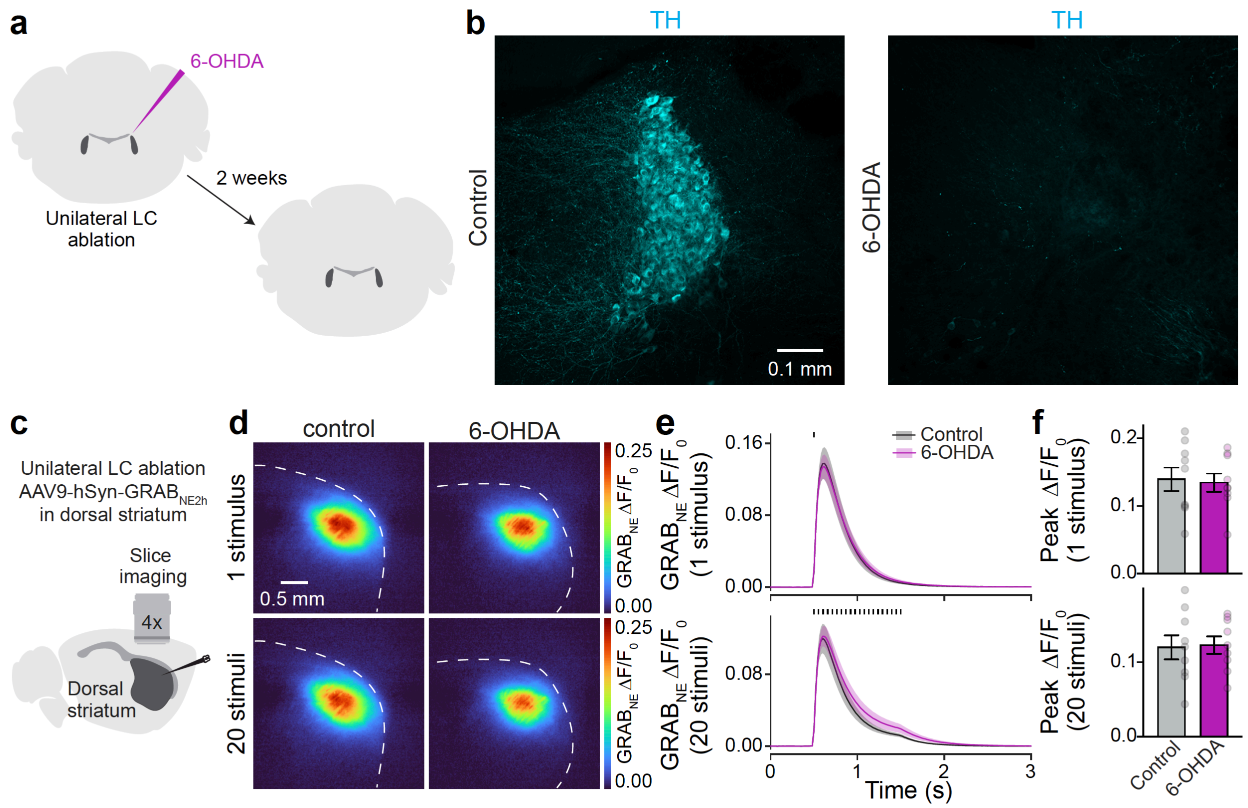

Supplemental figure 6. Striatal $GRAB_{NE}$ responses after 6-OHDA lesion of LC

a. Schematic of unilateral 6-OHDA ablation of LC followed by assessment with confocal microscopy.

b. Confocal images of LC after staining with TH antibodies; a representative image of 4 slices from 4 mice is shown.

c. Schematic of bilateral slice imaging in dorsal striatum expressing $GRAB_{NE}$ with unilateral 6-OHDA ablation of the right LC.

d-f. Representative images of peak $GRAB_{NE}$ fluorescence in response to 1 stimulus or 20 stimuli at 20 Hz in ipsilateral (6-OHDA) and contralateral (control) hemispheres (d) and quantification of $GRAB_{NE}$ $\Delta F/F_0$ time series (e) and peak $\Delta F/F_0$ (f); control 9 slices from 3 mice, 6-OHDA 9/3.

bioRxiv preprint doi: https://doi.org/10.1101/2024.11.23.624963; this version posted November 23, 2024. The copyright holder for this preprint (which was not certified by peer review) is the author/funder, who has granted bioRxiv a license to display the preprint in perpetuity. It is made available under aCC-BY 4.0 International license.

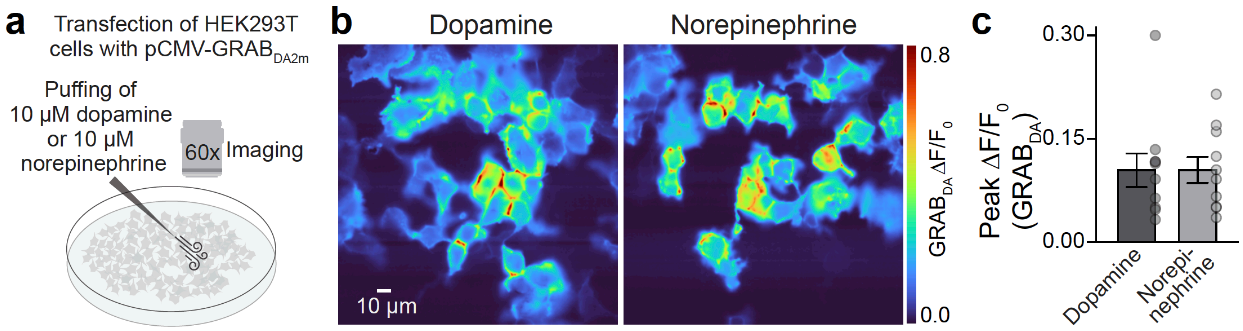

Supplemental figure 7. Assessment of GRAB$_{DA}$ fluorescence in HEK293T cells

a. Schematic of the experiment in which 10 μM of dopamine or norepinephrine were puffed onto HEK293T cells transfected with pCMV-GRAB$_{DA2m}$.

b, c. Representative peak GRAB$_{DA}$ fluorescence (b) and peak $\Delta F/F_0$ (c); 10 coverslips from 3 transfections each.

Data are mean ± SEM.

bioRxiv preprint doi: https://doi.org/10.1101/2024.11.23.624963; this version posted November 23, 2024. The copyright holder for this preprint (which was not certified by peer review) is the author/funder, who has granted bioRxiv a license to display the preprint in perpetuity. It is made available under aCC-BY 4.0 International license.

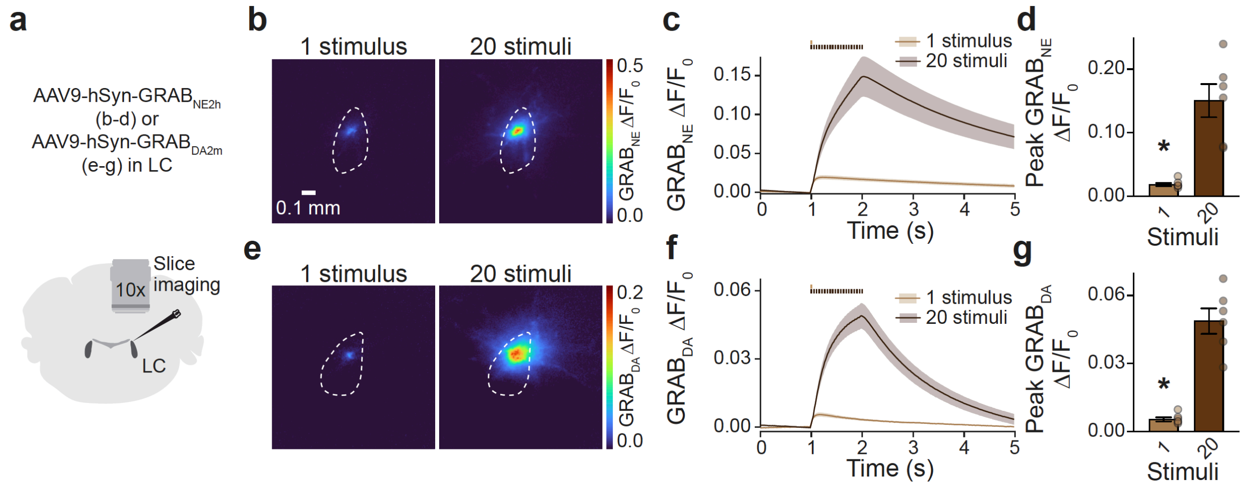

Supplemental figure 8. Norepinephrine and dopamine sensors in LC

a. Schematic of slice imaging in LC.

b-d. Representative $GRAB_{NE}$ peak fluorescence in response to 1 stimulus or 20 stimuli at 20 Hz in LC (b) and quantification of $\Delta F/F_0$ time series (c) and peak $\Delta F/F_0$ (d); 6 hemispheres from 3 slices from 3 mice.

e-g. As in b-d, but with $GRAB_{DA}$; 6/3/3.

Data are mean ± SEM; * < 0.05 as assessed by two-tailed Wilcoxon signed-rank tests in d and g.

bioRxiv preprint doi: https://doi.org/10.1101/2024.11.23.624963; this version posted November 23, 2024. The copyright holder for this preprint (which was not certified by peer review) is the author/funder, who has granted bioRxiv a license to display the preprint in perpetuity. It is made available under aCC-BY 4.0 International license.

Supplemental text. Studies with GPCR-based sensors in brain areas with dual dopamine and norepinephrine innervation

In the present study, we tested whether GPCR-based dopamine and norepinephrine sensors are specific for their corresponding transmitters in the brain. These sensors have been widely used in brain areas that are innervated by both norepinephrine and dopamine neurons and have been expressed in mice and rats.

Previous studies have used norepinephrine or dopamine sensors in the cortex[3,4,40–50] and in the cerebellum[51], brain areas in which norepinephrine innervation is typically higher in density compared to dopamine innervation[7,10] (Fig. 1a-c). Areas with dopamine and norepinephrine innervation densities that are likely in a similar range[52–56] have also been studied with these sensors, for example the hippocampus[5,57–60], the basolateral amygdala[3,49], the lateral hypothalamus[4,60–62], and the basal forebrain and thalamus[63]. Finally, dually innervated brain areas in which dopamine dominates over norepinephrine[55,64,65] have been characterized with the sensors as well, for example the ventral striatum[1–3,16,49,50,62,66–72] and the ventral tegmental area[49].

Overall, the sensor signals in these brain regions might be due to either dopamine or norepinephrine release. Without cell type- or projection-specific manipulations to remove either dopamine or norepinephrine innervation and/or secretion, it is difficult to conclude which transmitter is detected in dually innervated brain areas when GPCR-based sensors for dopamine or norepinephrine are used.
